# Oral dydrogesterone *vs.* vaginal progesterone capsules for luteal-phase support in women undergoing embryo transfer: a systematic review and meta-analysis

**DOI:** 10.5935/1518-0557.20180018

**Published:** 2018

**Authors:** Marina Wanderley Paes Barbosa, Natália Paes Barbosa Valadares, Antônio César Paes Barbosa, Adelino Silva Amaral, José Rubens Iglesias, Carolina Oliveira Nastri, Wellington de Paula Martins, Hitomi Miura Nakagawa

**Affiliations:** 1 Genesis - Centro de Assistência em Reprodução Humana, Brasília, DF, Brazil; 2 FMRP - USP - Faculdade de Medicina de Ribeirão Preto, Ribeirão Preto, SP, Brazil; 3 SEMEAR fertilidade, Ribeirão Preto, SP, Brazil

**Keywords:** dydrogesterone, IVF, luteal phase support, meta-analysis

## Abstract

**Objective:**

To identify, appraise, and summarize the evidence from randomized controlled trials (RCTs) comparing oral dydrogesterone to vaginal progesterone capsules for luteal-phase support (LPS) in women offered fresh or frozen embryo transfers following in vitro fertilization.

**Methods:**

Two independent authors screened the literature for papers based on titles and abstracts, then selected the studies, extracted data, and assessed the risk of bias. Dydrogesterone and progesterone were compared based on risk ratios (RR) and the precision of the estimates was assessed through the 95% confidence interval (CI).

**Results:**

An electronic search performed on June 7, 2017 retrieved 376 records, nine of which were papers deemed eligible and included in this systematic review and quantitative analysis. Good quality evidence indicates that oral dydrogesterone provided at least similar results than vaginal progesterone capsules on live birth/ongoing pregnancy (RR=1.08, 95%CI=0.92-1.26, I^2^=29%, 8 RCTs, 3,386 women) and clinical pregnancy rates (RR 1.10, 95% CI 0.95 to 1.27; I^2^=43%; 9 RCTs; 4,061 women). Additionally, moderate quality evidence suggests there is no relevant difference on miscarriage rates (RR=0.92, 95%CI=0.68-1.26, I^2^=6%, 8 RCTs, 988 clinical pregnancies; the quality of the evidence was downgraded because of imprecision).

**Conclusions:**

Good quality evidence from RCTs suggest that oral dydrogesterone provides at least similar reproductive outcomes than vaginal progesterone capsules when used for LPS in women undergoing embryo transfers. Dydrogesterone is a reasonable option and the choice of either of the medications should be based on cost and side effects.

## INTRODUCTION

Progesterone is needed to maintain early pregnancy, and progesterone supplementation in assisted reproduction technology (ART) cycles is a well-accepted procedure (Shapiro *et al*., 2014; Holmdahl *et al*., 1971; van der Linden *et al*., 2015). Luteal phase deficiency affects women undergoing ART for many reasons. The most widely accepted theory posits that luteal phase deficiency originates from premature negative feedback on LH secretion in the pituitary caused by supra-physiological levels of steroids during controlled ovarian stimulation (COS) sustained after oocyte aspiration by multiple corpora lutea (van der Linden *et al*., 2015; Fatemi 2009).

There is evidence that luteal phase support (LPS) with progesterone, human chorionic gonadotropin (hCG) or gonadotropin-releasing hormone (GnRH) agonists improves reproductive outcomes in women undergoing in vitro fertilization (IVF) (Shapiro *et al*., 2014; van der Linden *et al*., 2015; Fatemi *et al*., 2007; Vaisbuch *et al*., 2012; Merriam *et al*., 2015; Martins *et al*., 2016). Since hCG correlates with higher risk of ovarian hyper stimulation syndrome (van der Linden *et al*., 2015; Fatemi *et al*., 2007; Vaisbuch *et al*., 2012) and evidence of the benefits of GnRH agonists is still of very low quality (Martins *et al*., 2016), progesterone appears to be the best option for LPS.

Progesterone can be administered orally, intramuscularly, vaginally or rectally; all routes seem to present similar levels of efficacy (Shapiro *et al*., 2014; van der Linden *et al*., 2015; Vaisbuch *et al*., 2012; Merriam *et al*., 2015). First-pass metabolism substantially reduces the bioavailability of oral progesterone to <10% (Nahoul *et al*., 1993). Intramuscular progesterone has been associated with pain caused by daily injections, inflammatory response, and local abscess (van der Linden *et al*., 2015; Fatemi *et al*., 2007; Vaisbuch *et al*., 2012; Ghanem & Al-Boghdady, 2012). Although fewer adverse events are observed with the vaginal route (Maher *et al*., 2013), vaginal progesterone causes local irritation, discharge, and bleeding; it is also affected by coitus, since absorption is decreased after intercourse (Merriam *et al*., 2015; Ghanem & Al-Boghdady, 2012).

Dydrogesterone is a synthetic progestin with enhanced oral bioavailability, known for being highly selective for the progesterone receptor (Kupferminc *et al*., 1990; Domitrz *et al*., 1999). It is effective in treating reproductive disorders such as threatened abortion and recurrent pregnancy loss, and has also been investigated in the prevention of gestational hypertension and preterm birth (Carp, 2012; 2015; Hudic *et al*., 2016; Mohamad Razi *et al*., 2016). Dydrogesterone has also been described to provide similar reproductive results as vaginal progesterone (van der Linden *et al*., 2015; Barbosa *et al*., 2016). The oral route of administration is thought to be a more patient-friendly regimen that might improve compliance to treatment.

The objective of this systematic review and meta-analysis was to identify, appraise, and summarize the evidence from randomized controlled trials examining the efficacy, safety, and tolerability of oral dydrogesterone compared to vaginal progesterone capsules for LPS in women undergoing ART.

## MATERIAL AND METHODS

### Protocol and registration

The protocol of this review was registered at PROSPERO(CRD42017071571).

### Eligibility criteria

True randomized controlled trials (RCTs) comparing oral dydrogesterone to vaginal progesterone capsules for LPS in women undergoing ART (fresh or frozen embryo transfer following IVF/ICSI) were included. Quasi and pseudo-randomized trials were not included.

### Information sources

The following electronic databases were searched for RCTs: PubMed, Scopus, and Embase. The references of the included studies and related reviews were also hand-searched. 

### Search

The following terms were used, adjusting for each database as necessary: (IVF OR ICSI OR embryo OR blastocyst OR oocyte OR egg OR retrieval OR luteal) AND (dydrogesterone OR duphaston OR isopregnenone OR dehydrogesterone). There was no limitation regarding language, publication date or publication status.

### Study selection

Two authors (MWPB and CON) independently screened publications for titles and abstracts based on the pre-established inclusion criteria and checked for duplicates. The same authors examined the full text articles of the studies selected for inclusion in the review; a third author (WPM) was involved to solve disagreements as needed. The authors corresponded with original study authors to clarify study eligibility when required. 

### Data collection process

A data extraction form designed and pilot-tested by the authors was used to extract data from the included trials. In the event of studies with multiple publications, the main trial report was used as reference and additional details were supplemented from secondary reports. The authors corresponded with trial authors to get clarification when required. Data were extracted independently in a standardized manner by two authors (MWPB and CON); a third author (WPM) was involved to solve disagreements as needed.

### Data items

The following data were collected to characterize the included trials: authors; country; institution; funding sources; conflicts of interest; informed consent; approval by ethics committees; study design; period of enrollment; eligibility criteria; number of participants in each group at each stage; age and BMI (mean±SD) of participants; COS protocol and trigger; number of embryos transferred per woman; and implantation rate.

The primary outcomes for effectiveness were live birth and/or ongoing pregnancy rates, while the primary outcome for adverse effect was dissatisfaction. Ongoing pregnancy was used a surrogate indicator of live birth in trials not reporting the latter. Ongoing pregnancy was defined as evidence of fetal cardiac activity on ultrasound examination after 10-12 weeks of gestation (Daya, 2003). Ongoing pregnancy was calculated as the number of clinical pregnancies minus the number of miscarriages in the trials in which it was not described. Secondary outcomes were clinical pregnancy; miscarriage per clinical pregnancy (single fetal demise in twin or triplet pregnancies was not counted as miscarriage); and any reported side effects. 

Additional unreported data were collected from the authors of the studies. Where data could not be obtained, clinical pregnancy (and subsequent miscarriage or live birth) was assumed not to have occurred in women with cycle cancellation. No assumption was made for women lost to follow up for other reasons.

### Risk of bias in individual studies

Two authors (MWPB and CON) independently assessed the risk of selection bias (random sequence generation and allocation concealment); performance bias (blinding of participants and personnel); detection bias (blinding of outcome assessors); attrition bias (incomplete outcome data); reporting bias (selective outcome reporting), and other potential sources of bias (e.g.: difference in the number of embryos transferred, age of participants, co-interventions, early stopping). A third author (WPM) was involved to solve disagreements as needed. The Cochrane Collaboration criteria for judging risk of bias was used in this review (Higgins & Green, 2011): the trials were assigned 'low', 'high' or 'unclear' risk of bias. Blinding was not considered as a factor likely to affect the risk of performance and detection bias on reproductive outcomes, but it might be detrimental to the evaluation of participant satisfaction with treatment, since the main adverse effects related to the route of drug administration.

### Summary measures

Dichotomous variables were expressed as risk ratios (RR) and the precision of the estimates was evaluated by the 95% confidence interval (CI). The clinical relevance of all comparisons was assessed based on the precision of the estimates. A random effects model was used to address the differences in true effect size across studies, since doses were different. The random effects model also incorporated the heterogeneity observed among studies and thus produced more conservative confidence intervals (Higgins & Green, 2011).

### Summary of results

Review Manager 5.3.5 (Copenhagen: The Nordic Cochrane Centre, The Cochrane Collaboration, 2014) was used to combine the results comprised in the meta-analysis. The I_2_ index was used to assess heterogeneity. Increases in the risk of positive (e.g.: live birth) or negative (e.g.: miscarriage) outcomes in the meta-analysis were plotted to the right of the centerline, while decreases in the risk such outcomes were plotted to the left of the centerline. Since one multi-arm study was included, we were careful not to double count controls.

### Risk of bias across studies

In view of the difficulty detecting and correcting for publication bias and other reporting biases, the authors aimed to minimize the potential impact by performing a comprehensive search for eligible studies and by preventing the duplication of data. Additionally, a funnel plot was used to assess the presence of small-study effects suggestive of publication bias.

### Additional analyses

Sensitivity analysis was performed for primary outcomes to verify whether the conclusions would have been different if eligibility was restricted to studies at low risk of bias. 

### Overall quality of the body of evidence

A table was generated to summarize the review findings. The quality of the evidence for the main outcomes was evaluated following the Grading of Recommendations Assessment, Development and Evaluation (GRADE) Working Group recommendation (Guyatt *et al*., 2011): the limitations of included studies, inconsistency of effect, imprecision, indirectness, and risk of publication bias were considered. 

The quality of the evidence was graded in the following levels (Balshem *et al*., 2011): High quality = We are very confident that the true effect lies close to the effect observed in this review; Moderate quality = We are moderately confident in the effect estimate: the true effect is likely to be close to the effect observed in this review, but it might be substantially different; Low quality = Our confidence in the effect estimate is limited: the true effect may be substantially different from the effect observed in this review; Very low quality = We have very little confidence in the effect estimate: the true effect is likely to be substantially different from the effect observed in this review.

## RESULTS

### Study selection

**Figure 1:**
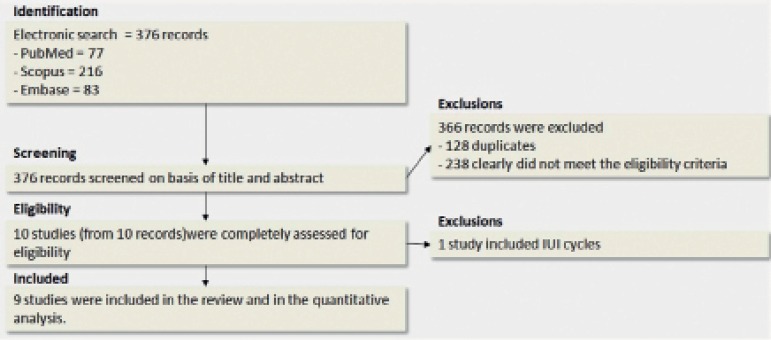
Flowchart of study selection.

An electronic search run in June 7, 2017 retrieved 376 records (PubMed = 77; Scopus = 216; Embase = 83). Additional papers hand-searched from the references of the included studies or related reviews were not included. Three hundred and four papers were excluded after their titles and abstracts were read: 128 were duplicates and 238 clearly did not meet the eligibility criteria. Ten studies were further examined for eligibility: one study was excluded because it compared dydrogesterone with vaginal progesterone capsules for luteal support in IUI cycles (Khosravi *et al*., 2015). Nine studies were included in our quantitative analysis (Chakravarty *et al*., 2005a; Ganesh *et al*., 2011; Patki & Pawar, 2007; Rashidi *et al*., 2016; Saharkhiz *et al*., 2016; Salehpour *et al*., 2013; Tournaye *et al*., 2017; Zarei *et al*., 2017; Zargar *et al*., 2016); four of the nine studies had groups given medication other than oral dydrogesterone and vaginal progesterone capsules: vaginal progesterone gel (Ganesh *et al*., 2011); intramuscular progesterone (Rashidi *et al*., 2016; Zargar *et al*., 2016); dydrogesterone combined with either GnRH agonist or hCG (Zarei *et al*., 2017). The individuals in these groups were not included in the quantitative analysis. Figure 1 shows the study flow diagram.

### Study characteristics

The characteristics of the nine parallel studies included in the quantitative analysis are reported in Table 1. One study was held in two centers (Saharkhiz *et al*., 2016), one in 38 different sites (Tournaye *et al*., 2017), and the remaining seven were carried out in single centers. Five studies were conducted in Iran (Rashidi *et al*., 2016; Saharkhiz *et al*., 2016; Salehpour *et al*., 2013; Zarei *et al*., 2017; Zargar *et al*., 2016), three in India (Chakravarty *et al*., 2005a; Ganesh *et al*., 2011; Patki & Pawar, 2007), and one in multiple centers in Austria, Belgium, Germany, Finland, Israel, Russia, and Spain (Tournaye *et al*., 2017). Despite attempts to contact the authors of all studies, additional details were collected from only one study (Saharkhiz *et al*., 2016). All studies were published as full articles. Only patients using vaginal progesterone capsules were used in the comparisons. 

**Table 1:** Characteristics of the included studies.

Study	Country	Period of enrollment	Eligibility criteria	Controlled ovarian stimulation	Intervention	Control	Study size*	Age*	BMI*
Chakravarty *et al.*, 2005a	India	Jan-2002 to Jun-2003	Women aged 25–42 years undergoing IVF/ICSI treatment (fresh cycles) with normal endometrial thickness (7–12mm) on the day of embryo transfer and no demonstrable endometrial disease	Long GnRH agonist using rFSH 150-300 IU/Day, triggering with hCG 10,000 IU IM	Dydrogesterone 20mg/day	VP capsule 600mg/day	N=430 (79 vs. 351)	<35 years: 47% vs. 49%; 35-40 years: 33% vs. 40%;> 40 years: 20% vs. 11%	NR
Ganesh *et al.*, 2011	India	NR	Normoprolactinemic euthyroid women aged 23-42 years with a history of tubal factor infertility, male factor infertility, idiopathic infertility, endometriosis-related infertility, and ovulatory disorders	Short GnRH agonist cycle, using rFSH 150-300 IU/Day, triggering with hCG 10,000 IU IM	Dydrogesterone 20mg/day	VP capsule 600mg/day	N=881 (422 vs. 459)	NR	NR
Patki & Pawar, 2007	India	Jan-2004 to Dec-2005	NR	Long GnRH agonist cycle, no other details	Dydrogesterone 30mg/day	VP capsule 600mg/day	N=675(366 vs. 309)	NR	NR
Rashidi *et al.*, 2016	Iran	Jan-2015 to May-2016	Patients undergoing FET with embryos left from past fresh or frozen cycles, canceled previous cycles (poor endometrium or ovarian hyper stimulation syndrome) or candidates for embryo donation.	NR	Dydrogesterone40mg/day	VP capsule800mg/day	N=120(60 vs. 60)	31.7±6.5 vs. 33.3±5.7	65.3±7.0 64.8±9.7
Saharkhiz *et al.*, 2016	Iran	Apr-2014 to Jan-2015	Women aged 20-40 years, BMI between 18-30 kg/m^2^, no visible endometrial pathology	Long GnRH agonist cycle or GnRH antagonist cycle, no other details	Dydrogesterone 40mg/day	VP capsule 800mg/day	N=210 (96 vs. 114)	30.6±5.4 vs. 31.0±5.2	26.0±3.5 vs. 26.2±3.8
Salehpour *et al.*, 2013	Iran	May-2012 to Dec-2012	Euthyroid normoprolactinemic women <40 years with male factor infertility	Short GnRH agonist cycle, using rFSH 150-300 IU/Day, triggering with hCG 10,000 IU IM	Dydrogesterone 40mg/day	VP capsule 800mg/day	N=80(40 vs. 40)	29.4±5.3 vs. 31.8±6.1	24.2±3.0 vs. 24.2±3.9
Tournaye *et al.*, 2017	38 sites in Austria, Belgium, Germany, Finland, Israel, Russia and Spain	Aug-2013 to Mar-2016	Euthyroid normoprolactinemic women aged 18-42 years, BMI ≥18 to ≤30 kg/m^2^ undergoing IVF (fresh cycles), FSH ≤15 IU/L and estradiol <80 pg/mL in early follicular phase (Day 2–4), normal transvaginal ultrasound, with <3 unsuccessful IVF attempts or history of ≤3 miscarriages	NR	Dydrogesterone 30mg/day	VP capsules 600mg/day	N=1031 (520 vs. 511)	32.5±4.5 vs. 32.5±4.4	23.3±3.1 vs. 23.2±3.2
Zarei *et al.*, 2016	Iran	Dec-2014 to Mar-2015	Patients aged 20-40 years undergoing FET, with unexplained infertility, tubal factor infertility, mild male factor infertility, premature ovarian failure, polycystic ovarian syndrome (PCOS), endometriosis stage I or II, and normal uterine cavity	Oral estradiol 6mg/day, until endometrial thickness reached 8-14mm; after that, IM progesterone injections 100 mg/day for 3 days.	Dydrogesterone 20mg/day	VP capsules 800mg/day	N=222 (110 vs. 112)	32.9±5.1 vs. 33.5±5.2	NR
Zargar *et al.*, 2016	Iran	Apr-2014 to Mar-2015	Women aged <40 years, with infertility lasting for <5 years, with regular menstrual cycles, normal hormone levels, and normal transvaginal ultrasound	Triggering with hCG 10,000 IU IM; no more details	Dydrogesterone 30mg/day	VP capsules 800mg/day	N = 412 (212 vs. 200)	30.0±5.0 vs. 31.9±4.8	NR

NR = not reported; ART = assisted reproductive technology; FET = frozen embryo transfer; VP = vaginal progesterone;

* = study vs. control

NOTES: One study reported a conflict of interest  (Tournaye *et al.*, 2017); Two studies reported funding sources (Tournaye *et al.*, 2017; Zarei *et al.*, 2017); All studies obtained approval from ethics committees, and one study did not provide informed consent  (Chakravarty  *et al.*, 2005a); All were parallel studies; Two studies reported an mean of three embryos transferred (Ganesh *et al.*, 2011; Salehpour *et al.*, 2013), and one study reported an mean of two embryos transferred  (Rashidi *et al.*, 2016).

Participants: 4,061 women submitted to ART in nine studies were included; 1,905 were allocated to groups prescribed dydrogesterone for luteal phase supplementation, and 2,156 were allocated to groups on vaginal progesterone capsules. The eligibility criteria, and therefore the characteristics of the included participants, were different across studies and are reported on Table 1.

Interventions: The nine studies assessed the use of daily oral dydrogesterone in doses ranging from 20mg to 40mg versus vaginal progesterone capsules in doses ranging from 600 mg/day to 800 mg/day. 

Outcomes: Two of nine studies reported live births (Rashidi *et al*., 2016; Tournaye *et al*., 2017); 3/9 reported ongoing pregnancies (Chakravarty *et al*., 2005a; Saharkhiz *et al*., 2016; Zarei *et al*., 2017); 8/9 reported clinical pregnancies (Ganesh *et al*., 2011; Patki & Pawar, 2007; Rashidi *et al*., 2016; Saharkhiz *et al*., 2016; Salehpour *et al*., 2013; Tournaye *et al*., 2017; Zarei *et al*., 2017; Zargar *et al*., 2016); 7/9 reported miscarriages (Chakravarty *et al*., 2005a; Ganesh *et al*., 2011; Rashidi *et al*., 2016; Saharkhiz *et al*., 2016; Salehpour *et al*., 2013; Zarei *et al*., 2017; Zargar *et al*., 2016); 2/9 reported female patient dissatisfaction (Chakravarty *et al*., 2005a; Saharkhiz *et al*., 2016); and 3/9 reported side effects (Saharkhiz *et al*., 2016; Salehpour *et al*., 2013; Tournaye *et al*., 2017). In one study, the number of clinical pregnancies was assumed to be equal to the summation of ongoing pregnancies and miscarriages (Chakravarty *et al*., 2005a). In three studies, the number of ongoing pregnancies was assumed to be equal to the number of clinical pregnancies minus miscarriages (Ganesh *et al*., 2011; Salehpour *et al*., 2013; Zargar *et al*., 2016).

### Risk of bias within studies

Six studies described adequate methods of randomization (Ganesh *et al*., 2011; Rashidi *et al*., 2016; Saharkhiz *et al*., 2016; Salehpour *et al*., 2013; Tournaye *et al*., 2017; Zarei *et al*., 2017; Zargar *et al*., 2016) and two studies did not report the method used (Chakravarty *et al*., 2005a; Patki & Pawar, 2007). Six studies described allocation concealment through sealed envelopes (Ganesh *et al*., 2011; Rashidi *et al*., 2016; Saharkhiz *et al*., 2016; Salehpour *et al*., 2013; Tournaye *et al*., 2017; Zargar *et al*., 2016). One study blinded participants and care providers (Tournaye *et al*., 2017). In six studies outcome assessors were blinded to allocation (Ganesh *et al*., 2011; Rashidi *et al*., 2016; Saharkhiz *et al*., 2016; Salehpour *et al*., 2013; Tournaye *et al*., 2017; Zargar *et al*., 2016) and the remaining three studies did not report whether outcome assessors were blinded.

Saharkhiz *et al*., 2016 was judged to be at high risk of attrition bias, since 24/234 (10.3%) participants were excluded after randomization; loss to follow up was unbalanced between groups, with 21/117 (17.9%) participants in the dydrogesterone group and 3/117 (2.6%) in the progesterone group. The other eight studies were judged to be at a low risk of attrition bias. Five studies analyzed all randomized women (Chakravarty *et al*., 2005a; Ganesh *et al*., 2011; Patki & Pawar, 2007; Salehpour *et al*., 2013; Zargar *et al*., 2016). Rashidi *et al*. (2016) excluded one of 120 participants from the analysis because she failed to come to embryo transfer due to a car accident; this study was deemed to present low risk of attrition bias since the withdrawal rate was low. Tournaye *et al*. (2017) excluded 57/1031 (5.5%) participants after randomization; loss to follow-up was balanced between groups - 23/520 (4.4%) in the dydrogesterone group and 34/511 (6.6%) in the progesterone group - and the study was considered to present low risk of attrition bias. For the same reasons the study by Zarei *et al*., (2017) was assigned low risk of attrition bias: 22/222 (10%) participants were excluded after randomization, but loss to follow-up was balanced between groups, with 10/110 in the dydrogesterone group and 12/112 in the progesterone group. 

The study by Zargar *et al*. (2016) was judged to present high risk of selective reporting bias, as three outcomes described in the registered protocol were not reported (live birth, preterm delivery, and perineal irritation caused by vaginal progesterone). Four studies reported all outcomes described in the registered protocol (Rashidi *et al*., 2016; Saharkhiz *et al*., 2016; Salehpour *et al*., 2013; Tournaye *et al*., 2017) and the remaining four were not assessed as presenting selective reporting bias. 

One study was deemed at high risk of bias for containing a larger proportion of women aged 40+ years in the dydrogesterone group (Chakravarty *et al*., 2005a). There was no suspicion of other sources of bias in the other eight studies.

### Results of individual studies

**Figure 2:**
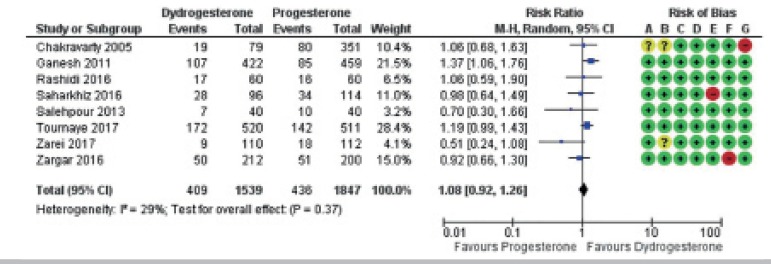
Forest plot for live birth/ongoing pregnancy. Risk of bias legend: A = Selection bias (random sequence generation); B = Selection bias (allocation concealment); C = Performance bias; D = Detection bias; E = Attrition bias; F = Reporting bias; G = Other biases.

**Figure 3:**
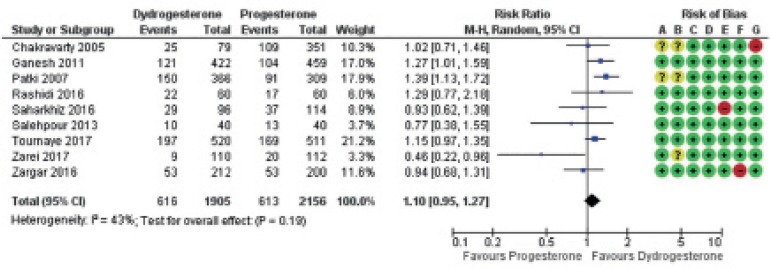
Forest plot for clinical pregnancy. Risk of bias legend: A = f4 Selection bias (random sequence generation); B = Selection bias (allocation concealment); C = Performance bias; D = Detection bias; E = Attrition bias; F = Reporting bias; G = Other biases.

**Figure 4:**
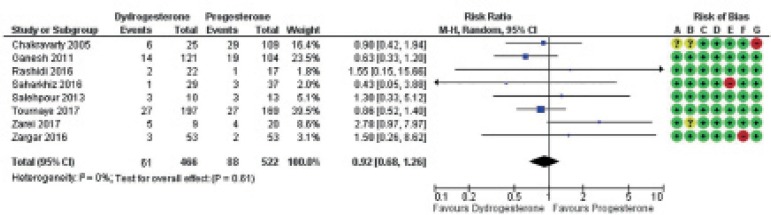
Forest plot for miscarriage. Risk of bias legend: A = Selection bias (random sequence generation); B = Selection bias (allocation concealment); C = Performance bias; D = Detection bias; E = Attrition bias; F = Reporting bias; G = Other biases.

Forest plots were used to show the results of each individual study and their respective possible biases (Figures 2-4)

### Summary of results

Live birth / Ongoing pregnancy (Table 2)

**Table 2:** Summary of findings.

	Absolute chance/risk (95% CI) ^a^		RR (95% CI)	N participants / studies	Interpretation	Quality of evidence
	**Vaginal progesterone capsules**	**Oral dydrogesterone**				
Live birth / Ongoing pregnancy	24%	25% (22-30%)	1.08 (0.92-1.26)	3,386 / 8	Dydrogesterone is better or no clinically relevant difference	High
Clinical pregnancy	28%	31% (27-36%)	1.10 (0.95-1.27)	4,061 / 9	Dydrogesterone is better or no clinically relevant difference	High
Miscarriage per clinical pregnancy	17%	16% (11-21%)	0.92 (0.68-1.26)	988 / 8	No clinically relevant difference	Moderate^1^
Dissatisfaction	One study showing a large reduction (RR=0.10, 95%CI=0.02-0.39) and the other study showing no significant difference (RR=1.19, 95%CI=0.46-3.04)					

All outcomes, except miscarriage, were analyzed per randomized women.

CI = confidence interval; RR = relative risk;

^a^ = The absolute risk in the Vaginal Progesterone group was determined as the mean risk in these groups; the absolute risk in the Oral Dydrogesterone group and its 95% CI was determined using the RR and its 95% CI;

^1^. Downgraded one level because of imprecision.

Overall, there was no evidence of relevant differences between oral dydrogesterone and vaginal progesterone on live birth/ongoing pregnancy rates: RR 1.08; 95% CI 0.92 to 1.26; I^2^=29%, 8 RCTs, 3,386 women; high quality evidence. In other words, considering a live birth/ongoing pregnancy rate of 24% in women using vaginal progesterone, this rate would be in the range of 22-30% in women using oral dydrogesterone. Sensitivity analysis excluding the three studies at high risk of bias did not change the estimate: RR 1.10; 95% CI 0.86 to 1.40; I^2^=48%, 5 RCTs, 2,334 women.

Clinical pregnancy (Figure 3)

Overall, there was no evidence indicating that clinical pregnancy was affected by the use of oral dydrogesterone versus vaginal progesterone capsules: RR 1.10, 95% CI 0.95 to 1.27; I^2^=43%; 9 RCTs; 4,061 women; high quality evidence. If 28% of the women using vaginal progesterone became pregnant, 27-36% of the women using oral dydrogesterone might also be clinically pregnant. Sensitivity analysis excluding the three studies at high risk of bias did not change the estimate: RR 1.08; 95% CI 0.86 to 1.36; I^2^ = 51%, 5 RCTs, 2,334 women.

Miscarriage (Figure 4)

Overall, there was no evidence indicating that miscarriage was affected by the use of oral dydrogesterone versus vaginal progesterone: RR=0.92, 95%CI=0.68-1.26, I^2^=6%, 8 RCTs, 988 clinical pregnancies; moderate quality evidence.

### Dissatisfaction

Two studies reported patient dissatisfaction with treatment (Chakravarty *et al*., 2005a; Saharkhiz *et al*., 2016) (26,30). Since the two studies were significantly heterogeneous (I^2^ = 91%), their results were not pooled together. Saharkhiz *et al*. (2016) reported no difference in dissatisfaction between groups: 8% in women using dydrogesterone *vs.* 7% in women using vaginal progesterone capsules; RR 1.19, 95% CI 0.46 to 3.04; 210 women. This study was deemed to be at high risk of bias. Chakravarty *et al*. (2005a) described a great benefit of dydrogesterone in reducing patient dissatisfaction: 3% in women using dydrogesterone *vs.* 26% in women using vaginal progesterone capsules; RR 0.10, 95% CI 0.02 to 0.39; 430 women.

### Side effects

Substantial heterogeneity (>50%) was found for all side effects reported by the three studies and therefore the results were not pooled together. While two studies did not describe differences in reported side effects between the two groups (Saharkhiz *et al*., 2016; Tournaye *et al*., 2017), one study showed that dydrogesterone was associated with more cases of vaginal bleeding (RR 2.38; 95% CI 1.18 to 4.78), nausea (RR 21.00; 95% CI 1.27 to 346.66), and abdominal pain (RR 13.00; 95% CI 0.76 to 223.33) when compared to vaginal progesterone capsules.

### Risk of bias across studies

**Figure 5:**
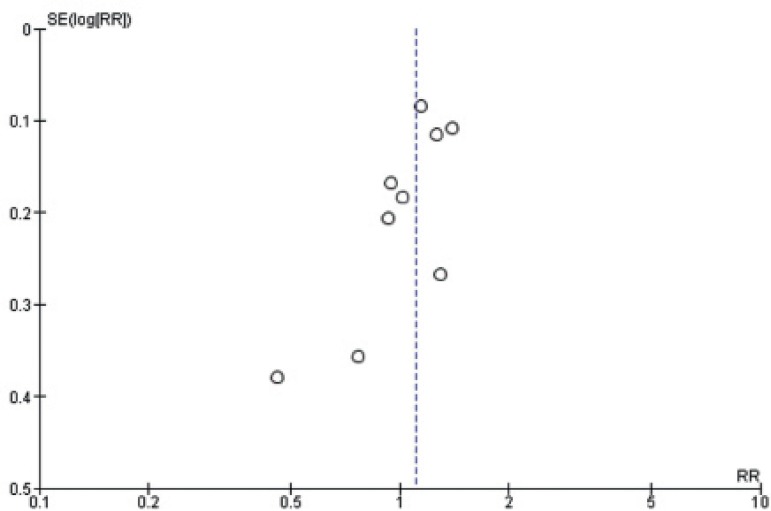
Funnel plot analysis for clinical pregnancy.

Although suboptimal, since fewer than 10 studies were included, the funnel-plot analysis for the only outcome reported in the nine studies - clinical pregnancy - was not suggestive of publication bias (Figure 5).

### Additional analysis

Sensitivity analysis was reported along with the synthesis of the results. 

## DISCUSSION

### Summary of the evidence

Nine studies were included in the comparison between oral dydrogesterone and vaginal progesterone capsules. Oral dydrogesterone was generally as effective as vaginal progesterone capsules for luteal phase support in women undergoing embryo transfers after IVF/ICSI. The assessment of patient dissatisfaction with treatment revealed an important inconsistency between the two studies reporting this outcome: one reported a significant difference favoring dydrogesterone (Chakravarty *et al*., 2005a) while the other found no differences between the regimens (Saharkhiz *et al*., 2016). Possible explanations for this discrepancy are the different doses of dydrogesterone and the potential differences in the characteristics of the two patient populations.

### Overall completeness and applicability of the evidence

Our findings were in agreement with the latest Cochrane review on the subject, which suggested a significant effect in favor of synthetic progesterone versus natural progesterone (van der Linden *et al*., 2015). Four studies were included in the comparison, three of which also included in our review (Chakravarty *et al*., 2005a; Ganesh *et al*., 2011; Patki & Pawar, 2007). The other study was not included in our review because it compared oral chlormadinone acetate to intramuscular progesterone (Iwase *et al*., 2008). Another recent review showed that dydrogesterone provides similar reproductive results when compared to vaginal progesterone (Barbosa *et al*., 2016). Seven studies were included in this review (Chakravarty *et al*., 2005a;b; *et al*., 2006; Ganesh *et al*., 2011; Patki & Pawar, 2007; Saharkhiz *et al.*, 2016; Salehpour *et al*., 2013), five of which were also included in our review (Chakravarty *et al*., 2005a; Ganesh *et al*., 2011; Patki & Pawar, 2007; Saharkhiz *et al*., 2016; Salehpour *et al*., 2013). Two of the studies were not included in our review because they were published as abstracts, thus yielding a high risk of bias to the comparison (Chakravarty *et al*., 2005 b; 2006). Four other studies were included in our review (Rashidi *et al*., 2016; Tournaye *et al*., 2017; Zarei *et al*., 2017; Zargar *et al*., 2016). One of these studies was sponsored by a pharmaceutical company (Tournaye *et al*., 2017), but its results were similar to the one described in other trials. The only difference between the study by Tournaye *et al*. (2017) and the others included was the double-blinding procedure, which in fact minimizes the risk of bias (Lexchin *et al*., 2003). With the addition of more studies in our review, and by excluding the abstracts, the authors believe that this review provides a robust body of evidence for the comparison between dydrogesterone and vaginal progesterone capsules for LPS in women undergoing embryo transfers.

In terms of dissatisfaction with treatment, our review included the same studies as the cited review (Barbosa *et al*., 2016). The discrepancy between the two studies in regards to this outcomes makes it difficult to draw firm conclusions. Different side effects were reported in three studies (Saharkhiz *et al*., 2016; Salehpour *et al*., 2013; Tournaye *et al*., 2017), and two of them did not report differences between the two groups (Saharkhiz *et al*., 2016; Tournaye *et al*., 2017). Additionally, a systematic review on the use of dydrogesterone for recurrent miscarriage found 13 studies reporting apparently minimal adverse effects (Carp, 2015).

### Limitations

The evidence available suffers from the limitations inherent to the included studies: five of the nine studies had high risk of bias in at least one domain; and the use of different doses in case and control groups along with different durations of LPS may have introduced some heterogeneity in the analysis. This issue was addressed with a random-effects model and by the incorporation of observed heterogeneity in the interpretation of the findings and in the assessment of the quality of the evidence.

### Quality of the evidence

The quality of the evidence was considered to be high for live birth/ongoing pregnancy and clinical pregnancy. It was downgraded one level for miscarriage because of imprecision: there was a relatively low number of events and a broad confidence interval.

## CONCLUSIONS

Oral dydrogesterone is as effective as vaginal progesterone capsules for luteal-phase supplementation in ART cycles. Oral dydrogesterone might be a good option in clinical practice, since oral administration is more patient-friendly than the vaginal route. The choice for either should be based mainly on availability, cost, and side effects.
